# Epigenetic and Molecular Alterations in Obesity: Linking CRP and DNA Methylation to Systemic Inflammation

**DOI:** 10.3390/cimb46070441

**Published:** 2024-07-13

**Authors:** Ciprian Cucoreanu, Adrian-Bogdan Tigu, Madalina Nistor, Radu-Cristian Moldovan, Ioana-Ecaterina Pralea, Maria Iacobescu, Cristina-Adela Iuga, Robert Szabo, George-Calin Dindelegan, Constatin Ciuce

**Affiliations:** 1 Department of General Surgery, “Iuliu Hatieganu” University of Medicine and Pharmacy, 400012 Cluj-Napoca, Romania; 2 Department of Translational Medicine, Research Center for Advance Medicine—MEDFUTURE, “Iuliu Hațieganu” University of Medicine and Pharmacy Cluj-Napoca, 400012 Cluj-Napoca, Romania; 3 Department of Proteomics and Metabolomics, Research Center for Advance Medicine—MEDFUTURE, “Iuliu Hațieganu” University of Medicine and Pharmacy Cluj-Napoca, 400012 Cluj-Napoca, Romania; 4 Department of Pharmaceutical Analysis, Faculty of Pharmacy, “Iuliu Haţieganu” University of Medicine and Pharmacy Cluj-Napoca, 400012 Cluj-Napoca, Romania; 5 Department of Anesthesia and Intensive Care, University of Medicine and Pharmacy “Iuliu Hatieganu”, 400012 Cluj-Napoca, Romania

**Keywords:** obesity, chronic inflammation, 5-methyl-Cytosine, epigenetics, C-reactive protein

## Abstract

Obesity is marked by excessive fat accumulation in the adipose tissue, which disrupts metabolic processes and causes chronic systemic inflammation. Commonly, body mass index (BMI) is used to assess obesity-related risks, predicting potential metabolic disorders. However, for a better clustering of obese patients, we must consider molecular and epigenetic changes which may be responsible for inflammation and metabolic changes. Our study involved two groups of patients, obese and healthy donors, on which routine analysis were performed, focused on BMI, leukocytes count, and C-reactive protein (CRP) and completed with global DNA methylation and gene expression analysis for genes involved in inflammation and adipogenesis. Our results indicate that obese patients exhibited elevated leukocytes levels, along with increased BMI and CRP. The obese group revealed a global hypomethylation and upregulation of proinflammatory genes, with adipogenesis genes following the same trend of being overexpressed. The study confirms that obesity is linked to systematic inflammation and metabolic dysfunction through epigenetic and molecular alterations. The CRP was correlated with the hypomethylation status in obese patients, and this fact may contribute to a better understanding of the roles of specific genes in adipogenesis and inflammation, leading to a better personalized therapy.

## 1. Introduction

Obesity is a key factor in numerous pathological conditions and represents one of the most challenging health issues in most developed countries. Since the World Health Organization declared obesity as a pandemic in 1997, the interplay between adipogenesis and health outcomes has gained significant scientific and clinical attention. Obesity is a chronic health disease characterized by excessive adipose tissue accumulation and etiologically complex, encompassing behavioral, environmental, genetic, and neurohormonal determinants. This multifactorial disorder needs a comprehensive and nuanced approach to both research and clinical management, reflecting its profound implications for public health [1,2].

Extensive research has been recently conducted to achieve a better understanding of the mechanisms underlying the pathogenesis of obesity and its associated comorbidities. Beyond its diverse etiologies, obesity is characterized at a molecular level as a chronic inflammatory state with the adipose tissue contributing to adipocyte dysfunction [3,4].

**Figure 1:**
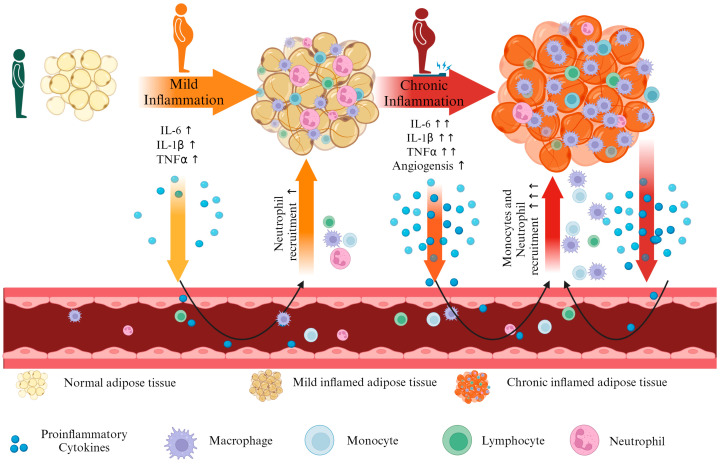
The link between inflammation and obesity. The illustration presents normal adipose tissue transformation into inflamed adipose tissue which produces cytokines (IL-6, IL-1β, TNFα) that recruit neutrophils into the adipose tissue. Prolonged inflammation led to increased cytokine production and monocytes recruitment in the inflamed adipose tissue, which will be translated into chronic inflammation in the adipocytes. The chronic inflammation creates a positive feedback loop that maintains the inflammatory status within the adipose tissue. The figure was created with Biorender.

Multiple studies have established that chronic inflammation within adipose tissue is fundamental to the organ dysfunction observed in obesity-related complications. Adipose tissue macrophages have a key role in the development of the inflammatory environment. Neutrophils, the most abundant leukocytes in the peripheral blood, are the first immune infiltrates in the adipose tissue. Upon activation, neutrophils release inflammatory mediators and recruit macrophages and other immune cells. As presented in Figure 1, the cellular activation amplifies the inflammatory state through the production of cytokines and chemokines, creating a systemic inflammatory condition characterized by an elevated level of tumor necrosis factor-alpha (TNFα), interleukin-1 (IL-1), interleukin-6 (IL-6), or interleukin-8 (IL-8) [5,6,7,8].

The adipose tissue dysfunction mechanism is also related to iron availability, which is an essential element in hemoglobin synthesis, enzyme activity, cell proliferation, and many other biological functions. Iron deficiency is particularly frequent in obese patients due to increased levels of circulating acute-phase reactant hepcidin and adiposity-associated inflammation. Recent studies highlighted the strong link between iron distribution and adipose tissue dysfunction, which involves various molecular pathways that modulate iron distribution and storage and impairs adipocyte function by enhancing inflammatory responses [9,10,11,12,13,14,15].

In addition to iron deficiency, the neutrophil/lymphocyte ratio (NLR) has been involved in the pathophysiology of obesity [16]. NLR is a known indicator of low-grade inflammation and has been linked to poor prognosis in various diseases. Given its association with systemic inflammation and metabolic dysfunction, NLR could serve as a predictor of perioperative outcomes in obese patients; thus, exploring NLR in the context of obesity and systemic inflammation could provide valuable insights into its utility as a prognostic tool in obesity-related complications [17,18,19].

The complex and interconnected nature of crucial signaling pathways that either facilitate or counteract obesity is a multifaceted phenomenon. Various pathways play a role in either promoting or hindering obesity, impacting different aspects such as appetite, thermogenesis, lipolysis, adipose tissue metabolism, glucose and fat homeostasis, adipogenesis, and energy expenditure. Major pathways include Mitogen-activated protein kinase (MAPK), Phosphoinositide 3-Kinsae/AKT (PI3K/AKT), Janus kinases/signal transducer and activator of transcription proteins (JAK/STATs), transforming growth factor beta (TGF-β), AMP-activated protein kinase (AMPK), and Wnt/β-catenin. These pathways are intertwined and can exert both activating and inhibitory effects. For example, the AMPK pathway not only directly affects lipolysis but also promotes insulin resistance and inflammation in adipose tissue. Similarly, the JAK/STAT pathway demonstrates anti-obesity effects by influencing thermogenesis, lipolysis, and hypophagia, while also contributing to the development of obesity through its impact on adipose tissue inflammation, mitochondrial dysfunction and insulin resistance, or tumorigenesis [20,21].

In the context of obesity, changes in the DNA methylation patterns may contribute to adipose tissue dysfunction and systemic inflammation. In other words, obesity as a multifactorial disease is influenced by the interaction of genetic predisposition and exposure to obesogenic environmental factors. Epigenetic changes may play a causative role in obesity by inducing inappropriate expression or silencing of obesity-related genes and regulatory sequences, thus leading to metabolic imbalances [22,23].

Obesity-induced changes in DNA methylation can affect genes that regulate iron metabolism and immune response. Changes in the methylation pattern, together with a systemic inflammation, may affect genes such as the fat mass and obesity-associated gene (*FTO*) which are involved in the metabolic and inflammatory pathways. These changes may be further correlated with low NLR and iron deficiency and prolonged systemic inflammatory status. By addressing the epigenetic modifications that contribute to *FTO* overexpression and other genes involved in adipogenesis (RNA methyltransferase complex methyltransferase-like protein (*METTL3*), YTH domain-containing reader proteins (*YTHDF1* or *YTHDF3*) together with the downstream effectors, it may be possible to mitigate some of the adverse metabolic and inflammatory outcomes associated with obesity [24,25].

Obesity is a major burden for the health condition by its derived comorbidities. One of the most common treatments in sustainable weight loss and comorbidity-associated risk decrease, especially for class III obesity, remains bariatric surgery [1,26]. Glucagon-like peptide 1 (GLP-1) and Glucagon-like peptide-1 receptor (GLP-1R) agonist therapy are newer proposed therapeutical options, being less invasive than bariatric surgery, with a mechanism of action which implies the modulation of insulin secretion, satiety, lipolysis, and even gastric motility, leading to significant weight loss [17,27]. On the other hand, the use of alternative anti-GLP-1 drugs is under continuous debate due to their possible side effects [28,29].

Alterations in DNA methylation may exacerbate metabolic dysfunction and lead to prolonged inflammation. General DNA hypomethylation can lead to increased expression of specific genes, which may be linked with better transcription of gene promoters, as it was seen in most of the studies focused on cancer [30,31].

Our study aims to elucidate the intricate relationship between DNA methylation, inflammation, and adipogenesis in obesity. A better understanding of the epigenetic mechanisms involved in obesity can open potential directions of research for better patient stratification and follow-up.

## 2. Materials and Methods

### 2.1. Patient Characteristics

The study was conducted at the Clinical County Emergency Hospital Cluj, Romania, during January 2022 and January 2024. Patients admitted for elective surgery were analyzed for eligibility. Cases of obese patients proposed for bariatric surgery were selected. Inclusion criteria were age above 18, consent to participate, body mass index above 30 kg/m^2^, and proposed for laparoscopic gastric sleeve surgery. Non-obese patients, referred to as controls, were adults (BMI < 25 kg/m^2^) admitted during the same period for elective procedures such as IPOM (intraperitoneal onlay mesh) repair, laparoscopic hiatal hernia repair, and open inguinal or umbilical hernia repair. Patients from both groups were excluded if they presented with a history of severe chronic diseases, neoplastic disease, autoimmune disease, iron supplementation, or gastrointestinal and genitourinary tract bleeding.

Baseline characteristics such as age, sex, and comorbidities were determined. Blood samples were obtained upon admission and sent for the following lab tests: full blood count, liver function tests, creatinine, urea and electrolytes, albumin, total proteins, lipid profile, and iron studies. Blood parameters were determined in the hospital’s laboratory as per standard protocols. The primary endpoint was the presence of elevated inflammatory state based on low-cost routine analysis.

Baseline characteristics, as well as lab results, were statistically analyzed. Based on data distribution, values were compared.

### 2.2. Sample Collection and Nucleic Acid Extraction

One PAXgene Blood RNA Tube (PreAnalytiX GmbH, a Qiagen/ BD Company, Zurich, Switzerland) containing RNA stabilization reagent mixed with 2.5 mL of peripheral blood and one 6 mL Tube coated with heparin were used for plasma storage, and the remaining blood was used for genomic DNA extraction. The fractioned samples were further stored at −80 °C until further use.

The DNA extraction was performed on whole blood stored at −80 °C using the PureLink genomic DNA kit with the whole-blood DNA extraction protocol provided by the manufacturer (Invitrogen, Waltham, MA, USA), starting from 200 µL of blood. The elution step was performed using ultrapure molecular-grade water (Invitrogen, Waltham, MA, USA) at a final volume of 50 µL and further stored at −80 °C until genomic DNA methylation analysis [32].

The RNA extraction was performed on blood collected on PAXgene tubes, starting with the first centrifugation step to obtain the pellet. Further, the pellet was washed with 4 mL of ultrapure water (Invitrogen, Waltham, MA, USA); then, the pellet was further processed with the PureLink RNA mini kit (Invitrogen, Waltham, MA, USA). The pellet was resuspended in 200 µL of phosphate buffer saline (PBS1X) and mixed with 200 µL of lysis buffer containing 2-mercaptoethanol. This was mixed well and centrifuged at 12,000× *g* for two minutes at room temperature. The supernatant was transferred into a new, sterile 1.5 mL tube and mixed with 200 µL of pure ethanol. This was mixed well and transferred over the filter column provided within the extraction kit. The sample processing followed the manufacturer’s protocol, with the final step involving sample elution that was performed by eluting the RNA with ultrapure water at a final volume of 35 µL. The samples were evaluated by the Nanodrop 2000 system (ThermoScientific, Waltham, MA, USA). The RNA samples were stored at −80 °C [33].

### 2.3. Global DNA Methylation and 5-mC Quantification by ELISA

Genomic DNA extracted from each patient was included in the global DNA methylation analysis using the colorimetric Methylated DNA Quantification Kit (Abcam, Cambridge, UK). For 5 methyl cytosine (5-mC) evaluation, control samples (*n* = 17) and patients with obesity (*n* = 31) were included, and the absorbance at 450 nm was used for the 5-mC quantification and compared with the standard curve, which was evaluated at 5 points (0.5 µg/mL 5-mC, 1 µg/mL 5-mC, 2 µg/mL 5-mC, 5 µg/mL 5-mC, and 10 µg/mL 5-mC). An amount of 200 ng of DNA was used for each sample, and the absorbance at 450 nm was measured by the TECAN Spark 10M (Tecan, Männedorf, Switzerland) [34]. The statistical evaluation was performed using GraphPad Prism (version 8) applying unpaired Mann–Whitney test (GraphPad Software, San Diego, CA, USA).

### 2.4. Gene Expression Analysis by RT-PCR

The RNA samples were treated to remove the DNA contamination using the Turbo DNA free Kit (Invitrogen, Waltham, MA, USA) following the manufacturer’s protocol: 10 µL of RNA mixed with 1.75 µL of Turbo DNA master mix. The samples were mixed and incubated at 37 °C for 30 min. After the incubation step, 2 µL of DNAse inhibitor was added, and the samples were vortexed. After 5 min of incubation at room temperature, the samples were centrifuged at 17,860× *g* for two minutes at room temperature, and 11 µL of the supernatant was collected in a new sterile tube. The resulting RNA samples were measured using the Nanodrop 2000 system (ThermoScientific, Waltham, MA, USA).

The treated RNA was further used for the cDNA synthesis, using the High-Capacity cDNA Reverse Transcription Kit (Applied Biosystems, Waltham, MA, USA). The same amount of RNA was used for the complementary DNA (cDNA) synthesis step, preparing all the samples at 1000 ng of RNA in maximum 10 µL of ultrapure water (Invitrogen, Waltham, MA, USA). The 10 µL of RNA were mixed with the cDNA master mix according to the manufacturer protocol, and the samples were incubated in a thermal cycler (Applied Biosystems, Waltham, MA, USA) as follows: 10 min at 25 °C, followed by 120 min at 37 °C and 5 min at 85 °C.

The resulting cDNA was further used for the gene expression analysis, using the PowerUp SYBR Green Master Mix (Applied Biosystems, Waltham, MA, USA) and following the manufacturer protocols and recommendations starting from 10 ng or cDNA per reaction for every gene included in the evaluation, as presented in Table 1. The RT PCR protocol included the 2 min uracil–DNA glycosylases (UDGs) activation step at 50 °C (1 cycle), followed by Dual-Lock DNA polymerase activation step at 95 °C (1 cycle), 40 cycles of denaturation at 95 °C for 3 s, and an anneal/extend step of 30 s at 60 °C. The RT PCR protocol was completed with a melting curve analysis to check the products for primer dimers or unspecific amplification. All the plates included a negative control (without DNA template), and all samples were evaluated in duplicates. The gene expression was evaluated by including the fold change values in GraphPad Prism (version 8), where the FC were transformed in Log2(Y), and the controls were compared with obese patients with an unpaired Mann–Whitney test. Only 14 samples from the obesity group and 15 from the control group were included in the evaluation based on the quality of the RNA after the DNAse treatment step [35].

### 2.5. Statistical Analysis

IBM SPSS version 26 was utilized to conduct statistical analysis. Variables were reported as mean ± standard deviation (SD) or percentages, where applicable. In cases of non-normal variables, the median ± and interquartile range were provided. The Shapiro–Wilk test was employed to assess the normal distribution of variables to determine the appropriate analysis method, whether parametric or nonparametric. Differences between groups were examined using either Student’s *t*-test or the Mann–Whitney *U* test, depending on the distribution of the data. Kendall tau correlation was used for measurement of the strength and direction of association between variables measured.

ROC analysis and graphical representations was performed using the Biomarker Analysis module of MetaboAnalyst 6.0 (accessed on 01 July 2024). Here, features with more than 50% missing values were removed, the remaining missing values were estimated using KNN featurewise estimation, and data were log10 transformed. The linear support vector machine algorithm was used for receiver operating characteristic curve (ROC) analysis on selected features.

The gene expression and 5-mC quantification were evaluated by including the fold change values in GraphPad Prism (version 8), where the FC were transformed in Log2(Y), and the controls were compared with obese patients with an unpaired Mann–Whitney test.

## 3. Results

### 3.1. Clinical Parameters

A total number of 121 patients were included in the study, with 65 included in the obese group (18 males and 47 females) and 56 in the control group (32 males and 24 females). The baseline characteristics are presented in Table 2.

Obese patients had significantly higher CRP levels compared to controls, indicating a higher baseline inflammatory status in obese patients compared to controls. Serum iron was significantly lower in obese compared to non-obese patients and may indicate a trend towards functional iron deficiency. The total leukocyte count was significantly increased in obese patients, with the total neutrophile, lymphocyte, and monocyte count following the same trend. In addition, the platelet number was significantly higher in the obese group. The neutrophil to leukocyte ratio was increased in the obese group compared to the control, but without statistical significance.

### 3.2. The DNA Methylation Status

**Figure 2:**
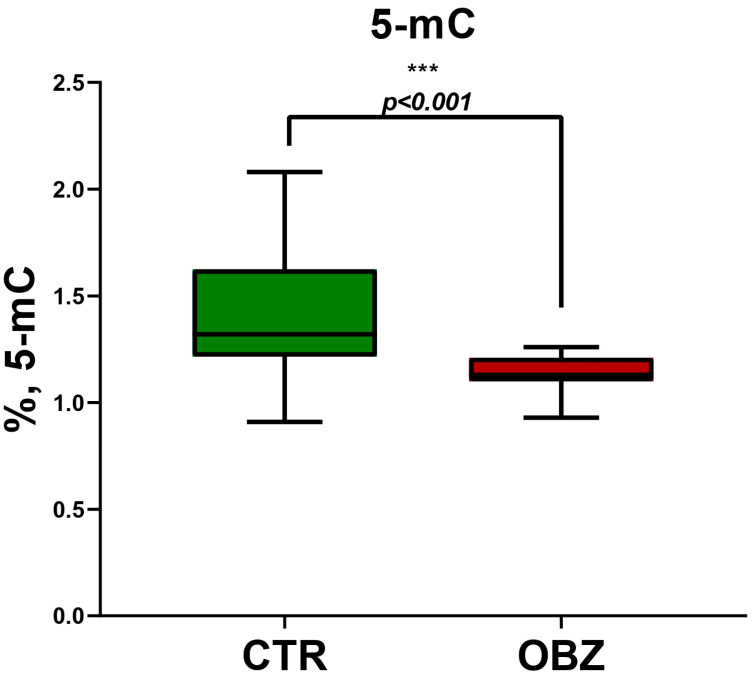
Global DNA methylation. The 5-mC percentages in obesity group (OBZ, *n* = 31) in comparison to healthy donors (CTR, *n* = 17). Statistical evaluation was performed using GraphPad Prism (version 8) applying unpaired two-tailed *t*-test with Welch’s correction *p* < 0.001 ***.

The genomic DNA methylation status was evaluated in control samples (*n* = 17) and samples from the obesity group (*n* = 31) by measuring the total amount of 5-methyl cytosine by the colorimetric method. The samples (*n* = 48) included in the assay were eligible for the 5-mC measurement and had a good quality of DNA. The average percent of 5-mC was 1.24%, with the highest methylation value of 2.08% in a sample from the control group, while the lowest methylation value was 0.91%, in a sample from the obesity group. The average methylation between the control and the obesity group was compared, as presented in Figure 2, and the difference between the two groups was statistically significant, with a *p* < 0.001. The results for the obesity group displayed more constant values than in the control, where the distribution was spread from less than 1% to 2.08% of 5-mC.

### 3.3. Gene Expression Analysis

**Figure 3:**
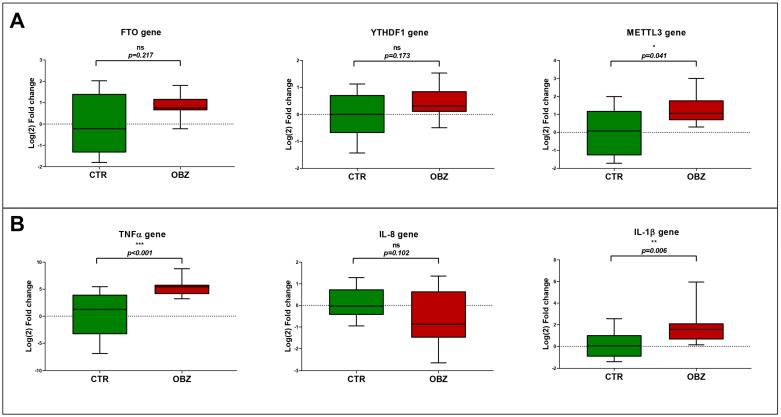
(**A**) Gene expression evaluation for the genes involved in adipogenesis and methylation pathways. *FTO*, *METTL3*, and *YTHDF1* are involved in the adipogenesis bioprocesses and modulate the nucleic acids methylation. (**B**) Gene expression evaluation for the genes involved in inflammatory processes. *TNFα*, *IL-1β*, and *IL-8* are involved in systemic inflammation and considered representative biomarkers. Obesity group (OBZ, *n* = 14) in comparison to healthy donors (CTR, *n* = 13). Statistical evaluation was performed using GraphPad Prism (version 8) applying unpaired two-tailed *t*-test with Welch’s correction *p* < 0.05 *, *p* < 0.01 ** and *p* < 0.001 ***.

The hypomethylation displayed within the obesity group indicates that the normal physiological mechanisms in controlling adipogenesis may be disrupted; thus, the evaluation of adipogenesis-associated genes *FTO*, *METTL3*, and *YTHDF1* was performed on the 29 samples, which included control samples (*n* = 14) and samples in the obesity group (*n* = 15). The gene expression analysis included, first, the panel of three genes involved in the adipogenesis, and the results highlighted in Figure 3. In all three cases, the obesity group had an overexpression trend, with statistically significant overexpression of *METTL3* (*p* = <0.001) when compared with the control group. The overexpression indicates that the adipogenesis was significantly overstimulated, and according to the BMI average of the obesity group presented in Table 2, the gene expression of adipogenesis-associated genes do correlate with the physiopathological status of the patients.

As adipogenesis is stimulated in obese patients, the inflammatory status of the obese patients was evaluated. The gene expression analysis for three genes that encode key proinflammatory cytokines, *TNFα*, *IL-1β*, and *IL-8*, was evaluated by RT-PCR on the same 29 samples that were eligible for RT-PCR for adipogenesis-associated genes. The results showed that there is a statistically significant upregulation of the *TNFα* in the obese group compared to the control (*p* < 0.001), and the fold change distribution within the obese group was very compact, indicating that TNFα could be relevant for the inflammatory status of the obese patients. *IL-1β* was significantly overexpressed in obese patients compared to the control (*p* = 0.006), while in the case of *IL-8*, the overexpression was not statistically significant (*p* = 0.102). The three proinflammatory cytokines’ overexpression does correlate with the clinical evaluation of the obese patients, where C-reactive protein was significantly elevated, as presented in Table 2.

### 3.4. Evaluation of Clinical Parameters in Epigenetic Context

Using the clinical parameters obtained between the studied groups (see Table 2), the methylation, and gene expression status, we aimed to establish a predictive model for a better discrimination between patients, taking into consideration the chronic inflammatory status, the global DNA methylation, and gene expression.

The correlations between clinical and genetic parameters are presented in Figure 3A, which was generated based on the data presented in Supplemental Table S1 representing Kendall’s tau-b analysis.

**Figure 4:**
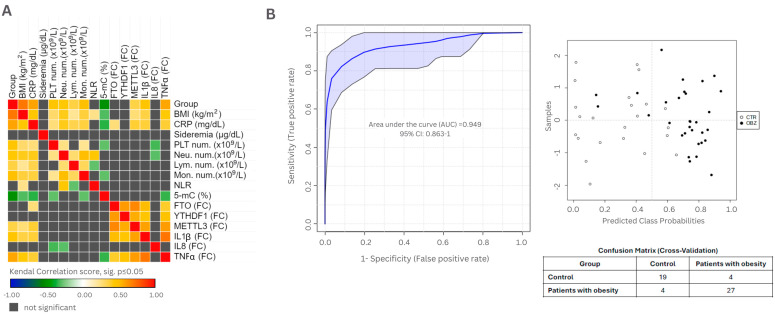
(**A**) Heat map representing the correlations between clinical and genetic parameters (Figure was generated with Morpheus, https://software.broadinstitute.org/morpheus (accessed on 14 June 2024)). (**B**) ROC analysis depicting the correlation between global DNA methylation and CRP level. Obesity group (OBZ, *n* = 31) in comparison to healthy donors (CTR, *n* = 23). PLTs = platelets; Neu. num.—neutrophil number; *Lym. num*.—lymphocyte number; *Mon. num*.—monocyte number; NLR—neutrophil-to-lymphocyte ratio; 5-mC—5 methyl-cytosine; *FTO* (FC)—*FTO* fold change; *YTHDF1* (FC)—*YTHDF1* fold change; *METTL3* (FC)—*METTL3* fold change; *IL1β* (FC)—*IL1β* fold change; *IL8* (FC)—*IL8* fold change; *TNFα* (FC)—*TNFα* fold change; AUC—area under the curve.

The most significant parameters were CRP and 5-mC. We evaluated the potential link between the baseline chronic inflammation and the level of methylated Cytosine. The ROC analysis presented in Figure 4B highlights a clustering of the obese patients, with an area under the curve (AUC) of 0.949, and shows a different distribution between the two studied groups, considering the elevated CRP and the hypomethylated status.

## 4. Discussion

Obesity is associated with fat accumulation in the adipose tissue. Increased fat accumulation led to an imbalance in the metabolic processes, triggering several dysregulations which are finally translated into energetic imbalance and mild, chronic, or systemic inflammation. Body weight is influenced also by external factors such as diet, physical activity, ambient temperature, lack of sleep, gut microbiota, different therapies, or even epigenetic factors [36]. As a general practice, BMI was considered one of the main indicators for clustering patients in normo-ponderal, overweight, or obese, which may indicate the risk of associated disease development, such as diabetes mellitus, cardiovascular disease, metabolic syndrome, or even some types of cancer. Nevertheless, BMI does not measure body fat directly, and it may not offer a precise risk stratification for obese patients. Thus, due to so many complications caused by increased BMI, obese patients need to act and lose weight either by diet, medication, or bariatric surgery followed by strict diet and improved lifestyle [37,38]. However, there are contradictions in evaluating the obesity burden for developing a morbid state. Caballero stated that the BMI stratification for overweight and obese patients may have a large heterogeneity when clustering the group with BMI between 25 and 30 [39]. According to some authors, the correlation between obesity-related metabolic disorders and BMI is relatively weak, and as a result, there is a large interest in the fields of genetic and epigenetic research [40]. DNA methylation may be triggered by adiposity and could be an indicator for obesity prediction and management. Our results indicate the link between 5-mC and CRP levels, which may prove relevant in differentiation of obesity grades.

Routine analysis performed on obese patients and control subjects revealed that the BMI was significantly increased in the obese group, as was expected, indicating that the obese patients have increased fatty mass and are prone to systemic inflammation and local monocyte and neutrophil infiltration in the adipose tissue. The excessive accumulation of fat in the adipose tissue, both by adipocytes’ hypertrophy or hyperplasia, causes stress and dysregulation, leading to inflammation, with the low-grade inflammation turning into chronic and, finally, systemic inflammation after a prolonged inflammatory phase [41,42,43]. In obese patients, stressed adipocytes secrete adipokines which recruit innate immune cells that maintain the inflammatory state through cytokine and chemokine production, thus generating systemic inflammation [44,45].

The total leukocyte count was significantly increased in obese patients compared to healthy donors (*p* < 0.001). Furthermore, the total count of monocytes was significantly increased in the obese group (*p* = 0.036), and the neutrophil total count followed the same trend (*p* < 0.001). The increased total leukocytes in the obese group could be responsible for the statistically significant increased level of CRP (*p* < 0.001). The role of neutrophil in acute inflammation was demonstrated in obesity. Neutrophils, as the primary effector cells of acute inflammation, are the first immune cells that infiltrate the adipose tissue. Then, they become activated and release proinflammatory factors that recruit macrophages and other immune cells such as B cells, T cells, or NK cells [7,46,47,48].

Monocytes are key players in metabolic syndrome and obesity [49]. Obesity is associated with an increased number of circulating monocytes, with an increased recruitment of monocytes in the inflamed adipose tissue [50]. The accumulation of proinflammatory macrophages in the adipose tissue, either via infiltration from circulation or local proliferation, sustains metabolic dysfunction and chronic or systemic inflammation [51]. As a result of previous studies, NLR was proposed as a prognostic factor for chronic inflammation and negative clinical outcome. Our results followed the trend of the literature, but without statistical significance, probably due to the low sample size. The chronic inflammatory environment and the activation of macrophages drives through iron disfunction which is linked with numerous series of clinical implications [14]. It was demonstrated that adipose tissue’s proinflammatory macrophages alter iron concentration and bioavailability, leading to exacerbated inflammation and worsening metabolic disfunction [52]. Some studies suggest that iron deficiency may be a trigger in the development of obesity and type 2 diabetes, impairing thermogenesis due to reduced binding of thyroid hormones to nuclear receptors and triggering impaired utilization of norepinephrine in tissues together with an impaired distribution of cortisol, which is translated to a reduced reactivity to stress [12]. We observed that the obese patient’s group was characterized by an increased total amount of leukocytes and monocytes, with a significantly decrease in iron availability (*p* = 0.012) compared to the control group. As bias, we must mention the heterogeneity of the analyzed data in our study, which does not include the complete count of ferritin and the coefficient of saturation of iron in transferrin.

Samocha-Bonet et al. stated that the platelet count was significantly increased with BMI [53]. Furthermore, metabolic syndrome seems to contribute to an increased number of platelets compared to the group of non-metabolic syndrome patients. There is an association between platelet count and body fat, mostly in the female group. Furthermore, the proinflammatory status could lead to a proatherogenic and prothrombotic landscape in obese patients, mostly due to proinflammatory cytokines [54,55]. According to Davi et al., android obesity is associated with lipid peroxidation and platelet activation, which are driven by inflammatory triggers related to abdominal adiposity [56,57]. Our results highlight the increased number of platelets in the obese patients’ groups (*p* = 0.026), demonstrating again the elevated thrombogenic risk associated with obesity. After clinic and genetic analysis, we found no correlation between hypomethylation status, inflammation, and platelet count.

DNA methylation is key to gene expression. Hypomethylated DNA can influence gene expression, generally by sustaining upregulation of different genes, including genes that encode proinflammatory molecules such as IL-1β or TNFα. The epigenetic processes affecting gene expression may contribute to the promotion of inflammation without inducing changes in the nucleotide sequences [58]. The methylation pattern can suffer modification due to many stimuli, with an important one being hypoxia. Hypoxia has several causes, one of them being increased fat deposits that are characteristic for obese patients. Many obese patients suffer from sleep apnea, which triggers inflammasome activation and maintains increased levels of proinflammatory signals. Thus, as a response to hypoxia in adipocytes, several gene promoters of proinflammatory cytokines are hypomethylated and upregulated [59]. In our study, we observed a global hypomethylated DNA where 5-mC levels were significantly lower in obese patients compared to the healthy donors. Furthermore, we detected that several adipogenesis-associated genes were significantly upregulated in obese patients and were related to an upregulation of proinflammatory genes such as *TNFα* and *IL-1β*. These results are correlated with the clinical evaluation of the obese patients, confirming the systemic inflammatory landscape within the obese group, and follow the previous reports in the literature. Ali et al. evaluated the methylation landscape in obese patients and observed that methylation is inversely correlated with BMI, blood pressure, serum IL-6, and CRP and positively correlated with HDL, folate, and vitamin B12 [60]. Other studies evaluated the link between DNA methylation and obesity-associated vitamin D deficiency, highlighting the fact that vitamin D deficiency may be responsible for obesity-related adipokine hypomethylation, vascular dysfunction, and inflammation [61]. Moreover, methylation status was demonstrated to be increased not only in obese patients but also in patients with diabetes and high BMI; thus, studies on the DNA extracted from adipose tissue could lead to novel potential biomarkers and genetic fingerprints in obese patients’ DNA [62].

However, obesity and obesity-related inflammation can be reversed with significant changes in terms of lifestyle and, in some cases, with medication or even surgical interventions. Bariatric surgery is becoming one of the most effective interventions to achieve sustained weight loss and to restore metabolic homeostasis and an overall healthy profile [63]. The established procedures, such as sleeve gastrectomy, bypass, biliopancreatic diversion, or gastric banding, have proven to be effective in losing weight and restoring the metabolic balance. Successful outcomes in bariatric surgery depend on the multidisciplinary approach, involving preoperative evaluation and patient selection, complex postoperative care, psychological counseling, and nutritional support consolidated with a regular follow-up to assure that the weight loss is sustained [63,64,65]. In this context, we furthermore highlight the importance of a better understanding of the molecular pattern of epigenetic changes in obesity and its clinical implications.

With a mean BMI around 45, the obese group also displayed elevated levels of CRP, indicating the systemic inflammatory status of the patients. The proinflammatory status, correlated with the high BMI, was confirmed by gene expression analysis, which showed overstimulated adipogenesis in the obese group compared to the controls. The *FTO* gene was evaluated by GWAS analysis (genome-wide association studies) and shown to be positively correlated with obesity; the studies were confirmed on murine models, where the *FTO* gene was overexpressed in obese-like models compared to the controls [25]. Moreover, *FTO* encodes an m6A RNA demethylase and is involved in several biological processes and metabolic pathways, and overexpression of the *FTO* gene was observed in type 2 diabetes. Moreover, *FTO* inactivation can lead to an increased protection from obesity, and its involvement in adipogenesis was confirmed by studies on animal models, which seem to lose weight when *FTO* is knocked out and reaches the control group parameters [66,67,68]. This *FTO* gene expression may lead to the discovery of novel genes involved in adipogenesis and lipolysis and may influence personalized therapy.

As obesity is one of the most concerning public health issues, the epigenetic evaluation of obese patients is a rising interest. The m6A RNA modification was detected to be a key regulator of obesity, with *YTHDF1* acting as a white adipose tissue metabolism regulator [69]. Like *YTHDF1*, the *METTL3* gene is involved in m6A methylation [70]; furthermore, *METTL3* is responsible for adipocyte differentiation and adipogenesis by modifying key genes leading to fat deposition [71,72]. The increased expression of the three genes, *FTO*, *METTL3*, and *YTHDF1*, with a high statistical significance, may indicate their potential involvement in the modulation of biological pathways that control adipogenesis.

The strong link between adipogenesis and inflammation is well known and may work as positive feedback loop with a never-ending activation through chemokines and cytokines. TNFα, IL-1β, IL-6, or IL-8 are proinflammatory cytokines that are considered obesity-linked inflammatory cytokines, predominantly in the abdominal fatty tissue. Moreover, TNFα and IL-6 can trigger the insulin receptors—starting insulin signaling cascades and the activation of JAK-STAT pathways—and are associated with an increased level of CRP [73,74]. Our results highlight the overexpression of TNFα and IL-1β in the obesity group, while the CRP was significantly higher on the obesity group compared to the controls; this correlates with the elevated BMI and low plasma iron distribution in the obese patients.

In obesity, the hypomethylation in the genomic DNA may indicate that several metabolic dysfunctions may occur due to the lack of gene transcription or an overexpression of genes involved in adipogenesis or inflammation. Gene expression analysis highlights the changes in the genome in patients characterized by a systemic inflammation and high BMI; furthermore, the 5-mC levels are lower than in healthy donors, indicating that the disrupted physiological functions may be triggered at the DNA level under epigenetic changes that are the root cause of too many diseases.

The unicentric design of the study and the low number of patients included are the main limitations of our study. The obese patients are clinically characterized by high BMI; elevated leukocytes, neutrophile, monocyte, and CRP; and lower iron plasmatic level, while the molecular aspects indicate a general hypomethylation of the genomic DNA, with significant overexpression of several genes that encode proinflammatory markers or proteins involved in adipogenesis or the nucleic acid methylation level.

## 5. Conclusions

Our study highlights the relation between DNA hypomethylation and inflammation in obese patients. By evaluating the molecular and epigenetic patterns and overviewing the link between inflammation markers (CRP, monocytes, leukocytes, neutrophils), gene expression analysis (*FTO*, *YTHDF1*, *METTL3*, *IL-1β*, *IL-8*, and *TNFα*), and global DNA methylation (5-mC), novel personalized therapies could be developed and could address more efficiently the obesity-related inflammatory and metabolic dysfunctions. Future comprehensive studies integrating genetic and epigenetic factors together with inflammatory status would be of interest and could reveal novel pathways in developing stratification models and possible personalized therapies for better outcomes in obesity management.

## Figures and Tables

**Table 1:** The nucleotide sequences for all primers that were used for gene expression analysis.

Primer	Sequence
*FTO* Forward	ACT TGG CTC CCT TAT CTG ACC
*FTO* Reverse	TGT GCA GTG TGA GAA AGG CTT
*METTL3* Forward	CAA GCT GCA CTT CAG ACG AA
*METTL3* Reverse	GCT TGG CGT GTG GTC TTT
*YTHDF1* Forward	GCA CAC AAC CTC CAT CTT CG
*YTHDF1* Reverse	AAC TGG TTC GCC CTC ATT GT
*TNFα* Forward	ATG GGC TAC AGG CTT GTC ACT C
*TNFα* Reverse	CTC TTC TGC CTG CTG CAC TTT G
*IL-1β* Forward	GTG CAG TTC AGT GAT CGT ACA GG
*IL-1β* Reverse	CCA CAG ACC TTC CAG GAG AAT G
*IL-8* Forward	GAG AGT GAT TGA GAG TGG ACC AC
*IL-8* Reverse	CAC AAC CCT CTG CAC CCA ATT T
*GAPDH* Forward	AGC CAC ATC GCT CAG ACA C
*GAPDH* Reverse	GCC CAA TAC GAC CAA ATC C

**Table 2:** Parameters of obese patients and controls on admission.

Variables	Controls	Patients with Obesity
Age (years; ±SD)	46.23 ± 15.21	42.41 ± 13.07
BMI (kg/m^2^)	23.18 ± 1.82	44.12 ± 5.95 ***
Median (p25–p75)	23.49 (22.06–24.65)	43.00 (39.20–47.43)
CRP (mg/dL)	0.28 ± 0.26	0.90 ± 0.68 ***
Median (p25–p75)	0.21 (0.10–0.39)	0.73 (0.45–1.24)
Sideremia (µg/dL)	86.96 ± 39.56	73.39 ± 27.47 *
Leucocytes (10^9^/L)	6.18 ± 1.28	8.91 ± 2.24 ***
Platelets (10^9^/L)	239.27 ± 61.86	284.49 ± 58.08 ***
Neutrophile count (10^9^/L)	4.16 ± 1.18	5.80 ± 1.79 ***
Lymphocyte count (10^9^/L)	1.86 ± 0.52	2.44 ± 0.73 ***
Median (p25–p75)	1.79 (1.46–2.21)	2.36 (1.83–2.71)
Monocyte count (10^9^/L)	0.38 ± 0.14	0.52 ± 0.17 ***
Median (p25–p75)	0.38 (0.30–0.45)	0.48 (0.40–0.61)
NLR	2.36 ± 0.82	2.55 ± 1.07
Median (p25–p75)	2.22 (1.68–3.03)	2.42 (1.76–3.06)

BMI = body mass index; CRP = C-reactive protein; NLR = neutrophil to lymphocyte ratio; significant differences regarding groups * *p* < 0.05, *** *p* < 0.001.

## Data Availability

Data are contained within the article.

## Supplementary Materials

The following supporting information can be downloaded at: https://www.mdpi.com/article/10.3390/cimb46070441/s1, Table S1: Correlations and Results for Kendall’s Tau-b.

## Abbreviation

BMI	Body mass index
CRP	C-reactive protein
DNA	Deoxyribonucleic acid
TNF α	Tumor necrosis factor alpha
IL-1B	Interleukin-1 beta
IL-6	Interleukin-6
IL-8	Interleukin-8
NLR	Neutrophil-to-lymphocyte ratio
FTO	Fat mass and obesity-associated gene
METTL3	RNA methyltransferase complex methyltransferase-like protein
YTHDF1	YTH domain-containing reader protein 1
YTHDF3	YTH domain-containing reader protein 3
GAPDH	Glyceraldehyde 3-phosphate dehydrogenase
IPOM	Intraperitoneal onlay mesh
RNA	Ribonucleic acid
5-mC	5-methyl-Cytosine
cDNA	complementary DNA
UDGs	Uracil–DNA glycosylases
ROC	Receiver operating characteristic curve
CTR	Control group
OBZ	Obesity group
AUC	Aria under the curve
PLT	Platelet
Neu num	Neutrophil number
Lym num	Lymphocyte number
Mon num	Monocyte number
FC	Fold change
SD	Standard deviation
GLP-1	Glucagon-like peptide-1
GLP-1R	Glucagon-like peptide-1 receptor
MAPKs	Mitogen-activated protein kinase
PI3K/AKT	Phosphoinositide 3-kinase/AKT
JAK/STAT	Janus kinases/signal transducer and activator of transcription protein
TGF-β	transforming growth factor beta
AMPK	AMP-activated protein kinase
